# Health-Related Behavioral Risk Factors and Obesity Among American Indians and Alaska Natives of the United States: Assessing Variations by Indian Health Service Region

**DOI:** 10.5888/pcd19.210298

**Published:** 2022-01-27

**Authors:** Guixiang Zhao, Jason Hsia, Alexander Vigo-Valentín, William S. Garvin, Machell Town

**Affiliations:** 1 Division of Population Health, National Center for Chronic Disease Prevention and Health Promotion, Centers for Disease Control and Prevention, Atlanta, Georgia; 2 Division of Policy and Data, Office of Minority Health, US Department of Health and Human Services, Rockville, Maryland

## Abstract

**Introduction:**

Health-related behavioral risk factors and obesity are linked to high risk for multiple chronic diseases. We examined the prevalence of these risk factors among American Indians and Alaska Natives (AI/ANs) compared with that of non-Hispanic Whites and across Indian Health Service (IHS) regions.

**Methods:**

We used 2017 Behavioral Risk Factor Surveillance System data from participants in 50 states and the District of Columbia to assess 4 behavioral risk factors (current cigarette smoking, heavy drinking, binge drinking, and physical inactivity) and obesity. We analyzed disparities in these risk factors between AI/AN and non-Hispanic White participants, nationwide and by IHS region, by conducting log-linear regression analyses while controlling for potential confounders.

**Results:**

Nationwide, crude prevalence of current smoking, physical inactivity, and obesity were significantly higher among AI/AN than non-Hispanic White participants. After adjustment for sociodemographic characteristics, AI/AN participants were 11% more likely to report current smoking (*P* < .05) and 23% more likely to report obesity (*P* < .001) than non-Hispanic White participants. These patterns persisted in most IHS regions with some exceptions. In the Southwest region, AI/AN participants were 39% less likely to report current smoking than non-Hispanic White participants (*P* < .001). In the Pacific Coast region, compared with non-Hispanic White participants, AI/AN participants were 54% less likely to report heavy drinking (*P* < .01) but 34% more likely to report physical inactivity (*P* < .05). Across IHS regions, AI/AN participants residing in Alaska and the Northern Plains regions had the highest prevalence of current smoking and binge drinking, and those in the Southwest and Pacific Coast regions had the lowest prevalence of current smoking. AI/AN participants in the Southwest region had the lowest prevalence of physical inactivity, and those in the Southern Plains region had the highest prevalence of obesity.

**Conclusions:**

The findings of this study support the importance of public health efforts to address and improve behavioral risk factors related to chronic disease in AI/AN people, both nationwide and among IHS regions, through culturally appropriate interventions.

**Summary**

**What is already known on this topic?**

Significant disparities in chronic disease prevalence and mortality rates exist among American Indians and Alaska Natives (AI/ANs), especially in comparison with non-Hispanic White people.

**What is added by this report?**

Population-based surveillance data demonstrated that, compared with non-Hispanic White participants, AI/AN participants experienced a greater burden of obesity and health-related behavioral risk factors, especially current cigarette smoking, nationwide and by Indian Health Service (IHS) region. We also found significant disparities in the AI/AN population across the 6 IHS regions.

**What are the implications for public health practice?**

Public health efforts to continue to address and improve these risk factors in the AI/AN population through culturally appropriate interventions are important, especially in IHS regions with a high prevalence of these factors.

## Introduction

Behavioral risk factors, such as cigarette smoking or commercial tobacco use, excessive alcohol use (eg, heavy and binge drinking), and sedentary lifestyle, as well as obesity are linked to increased risk for multiple chronic diseases including heart disease, hypertension, stroke, diabetes, and cancer (1–4). Improving lifestyle behaviors plays an important role in the prevention and control of these diseases (4–6).

American Indian and Alaska Native (AI/AN) people comprise a population with distinctive sociohistorical characteristics (7). In the US, the AI/AN population alone reached 3.7 million and the AI/AN population alone or in combination with other racial groups reached 7.1 million in 2020, which increased by 85.2% from 2010 to 2020 (8). The projected AI/AN population alone or in combination will reach 10.1 million in 2060 (8). AI/AN people have a higher burden of many chronic diseases including type 2 diabetes, hypertension, cardiovascular diseases, cancers, and mental illnesses (9–12). A national survey of the US adult population in 2014–2018 reported that, compared with all US adults, AI/AN adults had a significantly higher prevalence of having a severe disability (16.3% vs 8.9%), diagnosed hypertension (33.7% vs 28.7%), diagnosed diabetes (15.0% vs 8.6%), and multiple chronic conditions (31.9% vs 24.2%) (12). Significant disparities in all-cause or cancer mortality also existed among AI/AN individuals, especially in comparison with non-Hispanic White individuals (13). The high prevalence of these conditions may be linked to the health-related behaviors of AI/AN people; however, there is a growing consensus that historical trauma experienced by AI/AN communities as a consequence of the US federal policies of relocation, assimilation, and tribal termination are the underlying causes of many of these health behaviors and outcomes (14,15).

The health-related risk factors of AI/AN individuals have been reported for the periods of 1997–2000 (16,17), 2000–2006 (18), and 2000–2010 (19). These studies were conducted either among AI/AN people nationwide (16–18) or among the AI/AN population living in the Indian Health Service (IHS) Contract Health Service Delivery Areas (19). These studies have shown significantly higher prevalence of current smoking, excessive alcohol use, physical inactivity, and obesity among AI/AN people as compared with people from other racial and ethnic groups, especially non-Hispanic White people (16–19). However, due to the limited sample size of AI/AN people, these studies reported estimates based on multiple-year combined data (16–19). Consequently, the Office of Minority Health at the US Department Health and Human Services and the Centers for Disease Control and Prevention (CDC) launched an initiative in 2017 to oversample participants in states that have a high proportion of AI/AN people to improve understanding of the health status of AI/AN communities. The aim of this study was to expand knowledge of the health-related behaviors and obesity prevalence of AI/AN people. With an increased sample size of AI/AN individuals, we were able to examine these risk factors stratified by IHS region, which has not been done previously.

## Methods

### Study sample

The Behavioral Risk Factor Surveillance System (BRFSS) is a state-based, landline- and cellular-telephone survey of noninstitutionalized adults aged 18 years or older conducted annually to collect health information about participants’ general health status, health-related behavioral risk factors, health care access and use of preventive services, and chronic diseases and conditions in 50 states, the District of Columbia (DC), and participating US territories. Detailed information on BRFSS has been described elsewhere (20). BRFSS data provide compatible estimates for major health outcomes and health-related behaviors in comparison with other national surveys (21). 

In 2017, as part of a collaboration between CDC and the Office of Minority Health in an effort to improve understanding of the health status of AI/AN individuals, the BRFSS oversampled participants residing in 11 states (Alaska, Arizona, Minnesota, Montana, Nebraska, New Mexico, North Carolina, North Dakota, Oklahoma, South Dakota, and Wisconsin) that have a high proportion of AI/AN people. The oversample was to target specific banks of telephone numbers (with the same sampling frame of BRFSS) that were expected to yield a higher percentage of AI/AN respondents within these states. For this study, we analyzed data from 342,978 noninstitutionalized AI/AN (non-Hispanic) and non-Hispanic White participants residing in 50 states and the District of Columbia. The median response rate was 45.9% (ranging from 30.6% in Illinois to 64.1% in Wyoming) for the 2017 BRFSS (20).

### Measures

We examined the prevalence of obesity and 4 health-related behavioral risk factors: 1) current cigarette smoking, 2) heavy drinking, 3) binge drinking, and 4) physical inactivity. Participants were considered current smokers if they had smoked at least 100 cigarettes during their lifetime and were still smoking at the time the survey was conducted. Heavy drinking was defined as average daily consumption of more than 2 alcoholic drinks for men or more than 1 alcoholic drink for women during the previous 30 days. Binge drinking was defined as having 1 or more occasions of consuming 5 or more drinks for men or 4 or more drinks for women on an occasion during the previous 30 days. Physical inactivity was defined as not engaging in any moderately or vigorously intensive activities during leisure time over the previous 30 days. Obesity was defined as having a body mass index (BMI, kg/m^2^) of 30 or higher.

The 6 IHS regions were defined by state as previously reported (13,22): 1) Alaska; 2) Northern Plains (Illinois, Indiana, Iowa, Michigan, Minnesota, Montana, Nebraska, North Dakota, South Dakota, Wisconsin, and Wyoming); 3) Southern Plains (Oklahoma, Kansas, and Texas); 4) Southwest (Arizona, Colorado, Nevada, New Mexico, and Utah); 5) Pacific Coast (California, Idaho, Oregon, Washington, and Hawaii); and 6) East (the remaining 25 states and the District of Columbia).

Sociodemographic covariates were age (18–44, 45–64, and ≥65 y), sex (male, female), educational attainment (less than a high school diploma, high school diploma/GED, and more than a high school diploma), marital status (married, previously married [ie, divorced, widowed, or separated], and other [ie, never married or living with a partner]), and federal poverty level (FPL, <100%, 100%–199%, ≥200%, and unknown). The BRFSS protocol was approved by the CDC institutional review board and was determined to be exempt.

### Statistical analyses

Participants who responded “don’t know/not sure,” refused to answer, or had missing responses to any of the study covariates (except for income; the missing income/FPL was treated as an unknown group) were excluded from analysis, leaving 8,287 AI/AN and 334,691 non-Hispanic White participants eligible for analysis (N = 342,978). We calculated weighted prevalence with 95% CIs for health-related behavioral risk factors and obesity among AI/AN and non-Hispanic White BRFSS participants, nationwide and by IHS region. Adjusted prevalence ratios (APRs) and prevalence ratios with 95% CIs were estimated by conducting log-linear regression analyses with a robust variance estimator while adjusting for sociodemographic covariates, using non-Hispanic White participants (for comparisons between AI/AN and non-Hispanic White participants) or East region (for comparisons across IHS regions) as the reference groups. SAS-callable SUDAAN software (Research Triangle Institute) was used to account for the multistage, complex sampling design.

## Results

Of US adults, compared with non-Hispanic Whites, AI/AN participants were younger (mean age, 46.3 y vs 50.0 y), attained a lower level of education (46.3% vs 63.7% having more than a high school diploma), had lower proportions of being married (39.8% vs 55.7%), and had a lower income level (28.2% vs 49.5% with household income ≥200% FPL) (Table 1). Across 6 IHS regions, 2.8% of AI/AN participants lived in Alaska, 15.8% in the Northern Plains, 15.3% in the Southern Plains, 16.9% in the Southwest, 13.8% in the Pacific Coast, and 35.4% in the East regions; approximately 0.2%, 18.5%, 8.3%, 6.2%, 12.8%, and 53.9% of non-Hispanic Whites resided in these regions, respectively, in 2017. 

**Table 1:** Sociodemographic Characteristics of AI/AN and Non-Hispanic White Participants, by Indian Health Service Region, Behavioral Risk Factor Surveillance System, 2017^a^

Characteristic	US		Alaska		Northern Plains		Southern Plains		Southwest		Pacific Coast		East	
	AI/AN (n = 8,287)	NHW (n = 334,691)	AI/AN (n = 520)	NHW (n = 2,225)	AI/AN (n = 3,071)	NHW (n = 84,619)	AI/AN (n = 791)	NHW (n = 30,791)	AI/AN (n = 1,579)	NHW (n = 32,859)	AI/AN (n = 377)	NHW (n = 26,368)	AI/AN (n = 1,949)	NHW (n = 157,829)
Age, y														
18–44	47.4	39.6	50.7	47.6	51.9	41.1	51.4	39.7	54.6	41.1	37.0	38.6	44.1	39.1
45–64	35.5	35.2	36.1	35.2	36.0	35.1	32.5	36.0	33.3	33.5	34.6	34.8	37.9	35.3
≥65	17.1	25.2	13.2	17.2	12.1	23.7	16.1	24.3	12.1	25.3	28.4	26.7	18.0	25.6
Sex														
Male	50.0	48.6	49.8	53.5	49.7	48.7	48.1	49.0	46.9	49.3	49.9	49.2	52.4	48.2
Female	50.0	51.4	50.2	46.5	50.3	51.3	51.9	51.0	53.1	50.7	50.1	50.8	47.6	51.8
Education														
<HS	21.3	8.1	24.5	6.0	22.7	7.0	18.0	6.4	17.8	5.9	12.1	5.1	27.0	9.7
HS	32.4	28.2	41.0	25.0	32.3	30.1	34.4	25.6	35.1	22.9	30.7	22.4	30.2	30.0
>HS	46.3	63.7	34.5	68.9	44.9	62.9	47.7	67.9	47.1	71.2	57.2	72.5	42.8	60.4
Marital status														
Married	39.8	55.7	32.1	54.0	36.8	56.1	47.0	57.8	32.4	56.5	40.2	54.5	41.9	55.4
Previously married	28.0	20.9	24.1	18.6	23.6	19.5	28.3	21.8	23.6	20.8	32.1	21.5	30.6	21.1
Other	32.2	23.4	43.8	27.4	39.6	24.4	24.8	20.3	44.0	22.7	27.6	24.0	27.5	23.5
FPL, %														
<100	23.6	6.4	41.3	9.5	28.2	5.6	16.4	8.3	32.6	6.5	19.0	7.2	20.7	6.2
100–199	23.6	15.2	15.6	15.8	18.8	15.1	27.9	17.0	24.1	17.2	24.7	15.0	23.9	14.8
≥200	28.2	49.5	28.0	63.9	21.3	47.9	40.1	58.2	24.6	58.9	37.1	62.5	24.3	44.5
Unknown	24.6	28.8	15.1	10.8	31.7	31.3	15.5	16.6	18.8	17.4	19.1	15.2	31.0	34.5

Abbreviations: AI/AN, American Indians/Alaska Native; HS, high school diploma; FPL, federal poverty level; NHW, non-Hispanic White.

a Values are weighted percentages. IHS regions were defined as follows: 1) Alaska; 2) Northern Plains (Illinois, Indiana, Iowa, Michigan, Minnesota, Montana, Nebraska, North Dakota, South Dakota, Wisconsin, and Wyoming); 3) Southern Plains (Oklahoma, Kansas, and Texas); 4) Southwest (Arizona, Colorado, Nevada, New Mexico, and Utah); 5) Pacific Coast (California, Idaho, Oregon, Washington, and Hawaii); and 6) East (the remaining 25 states and DC).

The sociodemographic characteristics of AI/AN participants differed significantly across 6 IHS regions (Table 1). Notably, the Pacific Coast region had the highest percentage of people who were of AI/AN race or ethnicity and aged 65 years or older (28.4%) compared with the other regions (ranging from 12.1% to 18.0%) and had the highest percentage of AI/AN education level of more than a high school diploma (57.2%) compared with other regions (ranging from 34.5% to 47.7%). AI/AN participants residing in the Southern Plains region had the highest proportion of being married (47.0%) when compared with AI/AN participants living in other IHS regions (ranging from 32.1% to 41.9%) and had the highest proportion of a household income of 200% or higher of the FPL (40.1%) when compared with AI/ANs living in other IHS regions (ranging from 21.3% to 37.1%).

Nationwide, the crude prevalence of current smoking, physical inactivity, and obesity was significantly higher among AI/AN than non-Hispanic White participants (Table 2). The prevalence of heavy and binge drinking did not differ significantly between AI/AN and non-Hispanic White participants. After multivariable adjustment for sociodemographic characteristics, AI/AN participants remained 11% (*P* < .05) more likely to report current smoking and 23% (*P* < .001) more likely to report obesity than non-Hispanic White participants. These patterns persisted in most IHS regions, with some exceptions. In the Southwest region, although the crude prevalence of current smoking did not differ significantly between AI/AN and non-Hispanic White participants (15.7% vs 14.4%), after multiple adjustment for sociodemographic variables, AI/AN participants were 39% (*P* < .001) less likely to report current smoking than non-Hispanic White participants (Adjusted prevalence, 9.0% [95% CI, 7.5%–11.0%] vs 14.9% [95% CI, 14.3%–15.5%]). In contrast, in the Southern Plains region, although the crude prevalence of current smoking was significantly higher among AI/AN participants than non-Hispanic White participants (28.1% vs 17.0%, *P* < .001), the difference was attenuated after multiple variable adjustment (APR = 1.04 [95% CI, 0.72–1.53]). In the Pacific Coast region, the prevalence of current smoking did not differ significantly between the 2 groups; however, AI/AN participants were 54% (*P* < .01) less likely to report heavy drinking, 34% (*P* < .05) more likely to report physical inactivity, and 28% (*P* < .05) more likely to report obesity than non-Hispanic White participants after adjustment for sociodemographic characteristics.

**Table 2:** Crude Prevalence and Adjusted Prevalence Ratios for Health-Related Behavioral Risk Factors and Obesity Among AI/AN Participants Compared With non-Hispanic White Participants, Overall and by IHS Region, Behavioral Risk Factor Surveillance System, 2017

IHS region	AI/AN		Non-Hispanic White		APR (95% CI)
	n	%^a^ (95% CI)	n	%^a^ (95% CI)	
Overall					
Current smoking	7,877	28.9 (26.5–31.3)	323,059	17.1 (16.8–17.4)	1.11 (1.01–1.21)^b^
Heavy drinking	7,525	6.4 (5.2–8.0)	316,113	7.1 (6.9–7.3)	0.94 (0.75–1.16)
Binge drinking	7,539	16.4 (14.5–18.6)	316,380	17.8 (17.5–18.1)	0.95 (0.85–1.07)
Physical inactivity	7,063	32.1 (29.5–34.7)	302,520	27.4 (27.0–27.7)	1.00 (0.92–1.10)
Obesity	7,659	38.9 (36.4–41.4)	312,240	29.3 (29.0–29.7)	1.23 (1.15–1.31)^c^
Alaska					
Current smoking	488	43.2 (34.0–52.8)	2,166	16.8 (14.4–19.5)	1.56 (1.19–2.04)^c^
Heavy drinking	464	─^d^	2,129	9.0 (7.4–11.0)	1.05 (0.54–2.03)
Binge drinking	461	22.7 (15.3–32.3)	2,136	19.6 (17.1–22.4)	1.18 (0.81–1.72)
Physical inactivity	421	33.0 (24.4–43.0)	2,069	22.3 (19.8–25.1)	1.19 (0.85–1.68)
Obesity	482	44.3 (35.3–53.7)	2,139	32.7 (29.7–35.9)	1.39 (1.11–1.74)^e^
Northern Plains					
Current smoking	2,939	41.5 (36.1–47.0)	82,059	17.3 (16.7–17.8)	1.44 (1.25–1.67)^c^
Heavy drinking	2,809	9.1 (6.1–13.3)	80,545	7.1 (6.8–7.5)	1.29 (0.87–1.90)
Binge drinking	2,812	20.0 (15.8–25.1)	80,558	20.3 (19.8–20.9)	0.98 (0.79–1.21)
Physical inactivity	2,683	32.8 (27.8–38.1)	77,217	26.7 (26.1–27.2)	1.04 (0.89–1.21)
Obesity	2,848	37.8 (32.7–43.2)	79,317	31.3 (30.7–31.9)	1.14 (1.00–1.30)^b^
Southern Plains					
Current smoking	756	28.1 (21.6–35.6)	29,517	17.0 (15.6–18.6)	1.04 (0.72–1.53)
Heavy drinking	719	─^d^	28,672	7.2 (6.2–8.3)	0.95 (0.47–1.93)
Binge drinking	722	15.2 (10.0–22.3)	28,733	16.9 (15.5–18.4)	0.90 (0.62–1.29)
Physical inactivity	663	29.3 (22.6–37.0)	27,337	33.3 (31.4–35.2)	0.78 (0.58–1.04)
Obesity	722	46.3 (39.1–53.8)	28,421	31.6 (29.9–33.4)	1.37 (1.15–1.62)^c^
Southwest					
Current smoking	1,480	15.7 (13.0–18.7)	31,436	14.4 (13.9–15.0)	0.61 (0.50–0.74)^c^
Heavy drinking	1,413	5.7 (4.2–7.8)	30,871	6.5 (6.1–6.9)	0.88 (0.63–1.24)
Binge drinking	1,420	16.9 (14.0–20.2)	30,886	15.8 (15.2–16.4)	1.02 (0.84–1.23)
Physical inactivity	1,304	25.7 (22.5–29.2)	29,662	22.8 (22.2–23.5)	0.93 (0.82–1.07)
Obesity	1,435	39.6 (36.0–43.4)	30,740	24.5 (23.8–25.1)	1.53 (1.38–1.69)^c^
Pacific Coast					
Current smoking	361	17.6 (13.3–23.0)	25,432	13.2 (12.3–14.2)	0.94 (0.70–1.27)
Heavy drinking	351	3.6 (2.1–6.0)^f^	24,927	8.4 (7.7–9.2)	0.46 (0.27–0.78)^e^
Binge drinking	352	17.6 (11.1–26.7)^f^	24,923	17.8 (16.8–18.8)	1.10 (0.74–1.64)
Physical inactivity	328	31.8 (23.6–41.4)	23,784	18.9 (17.9–19.9)	1.34 (1.00–1.78)^b^
Obesity	351	35.7 (27.7–44.6)	24,663	25.6 (24.5–26.8)	1.28 (1.01–1.63)^b^
East					
Current smoking	1,853	32.7 (28.4–37.4)	152,449	18.2 (17.9–18.6)	1.17 (1.04–1.32)^e^
Heavy drinking	1,769	6.2 (4.1–9.1)	148,969	6.8 (6.6–7.1)	0.95 (0.63–1.42)
Binge drinking	1,772	14.3 (11.4–17.7)	149,144	17.3 (17.0–17.7)	0.84 (0.68–1.06)
Physical inactivity	1,664	35.8 (31.4–40.5)	142,451	29.2 (28.8–29.6)	1.02 (0.90–1.16)
Obesity	1,821	36.6 (32.4–41.1)	146,960	29.7 (29.3–30.1)	1.10 (1.00–1.24)^b^

Abbreviations: AI/AN, American Indian/Alaska Native; APR, adjusted prevalence ratio; IHS, Indian Health Service.

a Values are weighted percentages. APRs adjusted for age, sex, education, marital status, federal poverty level, and IHS region (for overall). IHS regions were defined as follows: 1) Alaska; 2) Northern Plains (Illinois, Indiana, Iowa, Michigan, Minnesota, Montana, Nebraska, North Dakota, South Dakota, Wisconsin, and Wyoming); 3) Southern Plains (Oklahoma, Kansas, and Texas); 4) Southwest (Arizona, Colorado, Nevada, New Mexico, and Utah); 5) Pacific Coast (California, Idaho, Oregon, Washington, and Hawaii); and 6) East (the remaining 25 states and DC).

b *P* < .05.

c *P* < .001.

d Estimates suppressed because of relative standard errors >30%.

e *P* < .01.

f Unstable estimates with relative standard errors ranging from 20% to ≤30%.

When comparing AI/AN participants across the IHS regions, after adjustment for sociodemographic characteristics, the prevalence of current smoking was highest among AI/AN participants residing in Alaska and the Northern Plains region and lowest among those in the Southwest and Pacific Coast regions (Table 3). Compared with AI/AN participants residing in the East region, those living in the Northern Plains region were 23% (*P* < .05) more likely to report current smoking and 34% (*P* < .05) more likely to report binge drinking. AI/ANs living in the Southern Plains were 29% (*P* < .01) more likely to report obesity; AI/ANs living in the Southwest were 53% (*P* < .001) less likely to report current smoking and 23% (*P* < .01) less likely to report physical inactivity; and AI/ANs living in the Pacific Coast region were 38% (*P* < .01) less likely to report current smoking, after adjustment for sociodemographic characteristics (Table 3).

**Table 3:** Adjusted^a^ Prevalence and APRs for Health-Related Behavioral Risk Factors and Obesity Among AI/AN Participants, by IHS Region, Behavioral Risk Factor Surveillance System, 2017

IHS region	Current smoking		Heavy drinking		Binge drinking		Physical inactivity		Obesity	
	% (95% CI)	APR (95% CI)	% (95% CI)	APR (95% CI)	% (95% CI)	APR (95% CI)	% (95% CI)	APR (95% CI)	% (95% CI)	APR (95% CI)
Alaska	39.1 (32.2–47.4)	1.22 (0.97–1.53)	9.4 (4.7–18.9)	1.57 (0.69–3.55)	21.3 (14.8–30.6)	1.47 (0.95–2.27)	33.1 (24.4–44.8)	0.96 (0.69–1.34)	42.6 (34.5–52.7)	1.17 (0.92–1.49)
Northern Plains	39.6 (34.6–45.3)	1.23 (1.03–1.47)^b^	9.0 (6.2–12.9)	1.49 (0.87–2.56)	19.5 (15.7–24.1)	1.34 (1.00–1.82)^b^	32.2 (27.7–37.4)	0.94 (0.77–1.14)	37.6 (32.9–43.0)	1.03 (0.87–1.23)
Southern Plains	29.3 (22.6–38.2)	0.91 (0.68–1.23)	6.7 (3.5–13.1)	1.12 (0.53–2.36)	15.1 (10.8–21.1)	1.04 (0.70–1.55)	31.8 (24.8–40.7)	0.93 (0.70–1.22)	47.1 (40.7–54.5)	1.29 (1.08–1.55)^c^
Southwest	15.2 (12.6–18.2)	0.47 (0.38–0.59)^d^	5.9 (4.2– 8.2)	0.98 (0.58–1.65)	15.8 (13.1–18.9)	1.09 (0.82–1.45)	26.3 (23.1–30.0)	0.77 (0.64–0.92)^c^	38.9 (35.4–42.8)	1.07 (0.92–1.24)
Pacific Coast	20.0 (15.2–26.3)	0.62 (0.46–0.84)^c^	4.0 (2.4– 6.7)	0.67 (0.35–1.28)	19.5 (13.5–28.0)	1.34 (0.88–2.05)	32.4 (24.3–43.3)	0.94 (0.69–1.29)	37.1 (29.4–46.7)	1.02 (0.79–1.31)
East	32.1 (28.3–36.5)	1 [Ref]	6.0 (4.0– 9.0)	1 [Ref]	14.5 (11.6–18.1)	1 [Ref]	34.4 (30.3–38.9)	1 [Ref]	36.4 (32.4–40.9)	1 [Ref]

Abbreviations: AI/AN, American Indian/Alaska Native; APR, adjusted prevalence ratio.

a Adjusted for age, sex, education, marital status, and federal poverty level. IHS regions were defined as follows: 1) Alaska; 2) Northern Plains (Illinois, Indiana, Iowa, Michigan, Minnesota, Montana, Nebraska, North Dakota, South Dakota, Wisconsin, and Wyoming); 3) Southern Plains (Oklahoma, Kansas, and Texas); 4) Southwest (Arizona, Colorado, Nevada, New Mexico, and Utah); 5) Pacific Coast (California, Idaho, Oregon, Washington, and Hawaii); and 6) East (the remaining 25 states and DC).

b *P* < .05.

c *P* < .01.

d *P* < .001.

## Discussion

Our results from a large, population-based survey demonstrated that AI/AN people nationwide were significantly more likely to report current smoking and having obesity compared with non-Hispanic White people. These patterns persisted in most of the IHS regions with a few exceptions: 1) AI/ANs were 39% less likely to report current smoking than non-Hispanic Whites in the Southwest region, and 2) the prevalence of current smoking did not differ significantly between the 2 groups in the Southern Plains and the Pacific Coast, after adjustment for sociodemographic variables. Moreover, we found that, in the Pacific Coast region, AI/AN people were 54% less likely to report heavy drinking but 34% more likely to report physical inactivity than non-Hispanic White people, which was not observed when analyses were conducted at the nationwide level. Furthermore, across IHS regions, AI/AN people residing in Alaska and the Northern Plains region had the highest prevalence of current smoking and binge drinking; AI/AN people in the Southwest and Pacific Coast regions had the lowest prevalence of current smoking. AI/AN people in the Southwest region also had the lowest prevalence of physical inactivity, and AI/AN people in the Southern Plains region had the highest prevalence of obesity, after adjustment for sociodemographic characteristics.

Our findings that AI/AN individuals are more likely than non-Hispanic White individuals to report current smoking and having obesity at the nationwide level are consistent with reports of previous studies. For example, 3 studies reported that the prevalence of current smoking and obesity were higher in AI/AN individuals than non-Hispanic White individuals during the periods of 1997−2000, 2000−2006, and 2000−2010 (16,18,19). For alcohol consumption, some inconsistent results were reported in the literature. An early study reported that the rates of alcohol-attributable deaths were higher among an AI/AN population than a White population, mainly resulting from alcohol-related causes that were acute (eg, hypothermia, alcohol poisoning) or chronic (eg, alcoholic psychosis, alcoholic liver disease) (23). Of note, these higher mortality rates were reported using data collected from 1999 to 2009 in IHS Contract Health Service Delivery Areas counties with an IHS patient registration database (23). However, several other studies based on nationwide surveillance data from the 1997–2000 and 2000–2006 BRFSS did not show significant difference in overall alcohol consumption between AI/AN and non-Hispanic White people (16,18). When analyses were stratified by sex, Steele et al reported the prevalence of heavy drinking among AI/AN men was significantly higher than among non-Hispanic White men (8.8% vs 6.7%), whereas the prevalence was lower among AI/AN women than among non-Hispanic White women (3.5% vs 5.3%) (18). Another study using data from the National Survey on Drug Use and Health also reported that Native American individuals had lower or comparable rates for several alcohol measures examined when compared with White individuals, which was in contrast to the “Native American elevated alcohol consumption” belief (24). Our findings further demonstrated that no significant difference in alcohol consumption between AI/AN and non-Hispanic White individuals existed, even after adjustment for sociodemographic characteristics. Similarly, no significant differences in heavy or binge drinking were observed among AI/AN individuals across the 6 IHS regions, except for AI/AN residents of the Northern Plains region, who were 34% more likely to report binge drinking than those residing in the East region. Future research should focus on evaluating the time trend of alcohol consumption among AI/AN individuals and further evaluate the long-term effects of alcohol use on mortality risk among both AI/AN and non-Hispanic White populations.

For physical inactivity, both Denny et al and Steele et al reported that AI/AN people had higher prevalence estimates of no leisure-time physical activity than non-Hispanic White people, using data from the 1997–2000 (32.5% vs 27.5%) and 2000–2006 (31.0% vs 21.5%) BRFSS (16,18). We did not observe significant differences in physical inactivity between AI/AN and non-Hispanic White people at the nationwide level after adjustment for sociodemographic characteristics. Nevertheless, our findings indicate that approximately one-third of AI/AN people remain physically inactive and one-sixth of AI/AN people engage in binge drinking. Given that these health-related behaviors are linked to increased risk of developing multiple chronic diseases and conditions (1–4), public health interventions aiming to improve the overall health of AI/AN people should still target these risk factors (ie, health-related behavioral risk factors and obesity) that put people at high risk for other health outcomes (25).

We did find significant regional variations in 2 aspects: 1) variations in the magnitude of the differences between AI/AN and non-Hispanic White participants regarding their health-related behaviors or obesity, overall and by IHS region, and 2) variations in health-related behaviors or obesity among AI/AN participants by IHS region, even after multivariable adjustment for sociodemographic characteristics. Across 6 IHS service regions, AI/AN participants were 10% (in East) to 53% (in Southwest) more likely to report obesity than non-Hispanic White participants. Although AI/AN participants were 17% (in East), 44% (in Northern Plains), and 56% (in Alaska) more likely to report current smoking than non-Hispanic White participants in those regions, the prevalence of current smoking did not differ significantly between the 2 racial groups in the Southern Plains and Pacific Coast regions. However, AI/AN participants residing in the Southwest region were 39% less likely to report current smoking than non-Hispanic White participants of this region. These findings are consistent with what has been reported previously (26,27). In our study, AI/AN people living in the Pacific Coast region seem to have unique characteristics, being less likely to engage in heavy drinking and more likely to be physically inactive compared with non-Hispanic White people of the same region.

Regional variations were particularly apparent within the AI/AN population. After adjustment for sociodemographic characteristics, AI/AN people residing in Alaska and the Northern Plains region had the highest prevalence of current smoking (39.1% and 39.6%, respectively), whereas AI/AN people in the Southwest and Pacific Coast regions had the lowest prevalence of current smoking (15.2% and 20.0%, respectively). AI/AN individuals in the Southwest region had the lowest prevalence of physical inactivity (26.3%); AI/AN individuals in the Southern Plains region had the highest prevalence of obesity (47.1%), and AI/AN individuals in the East region had the lowest prevalence of binge drinking (14.5%). Although specific reasons that contribute to the significant disparities across IHS regions remain unclear, these variations do suggest that some AI/AN communities may be more at risk than others; therefore, our findings could help prioritize public health intervention programs, health education, and resources from IHS and other government agencies focusing on at-risk AI/AN communities. Studies indicate that health-related behaviors are associated with self-rated health status (28) and health-related quality of life (29,30), as well as multiple chronic conditions (31,32). Disparities in health outcomes associated with the 5 risk factors we assessed will be evaluated further for the AI/AN communities.

Healthy People 2030 Objectives for health-related risk factors include 1) reduce current cigarette smoking in adults to 5.0% (TU-02), 2) reduce the proportion of people aged 21 years or older who engaged in binge drinking in the past month to 25.4% (SU-10), 3) reduce the proportion of adults who do no physical activity in their free time to 21.2% (PA-01), 4) increase the proportion of adults who do enough aerobic physical activity for substantial health benefits to 59.2% (PA-02), and 5) reduce the proportion of adults with obesity to 36.0% (NWS-03) (33). Our findings suggest that, for some of the risk factors (eg, current cigarette smoking, physical inactivity), greater efforts are needed to achieve those objectives for both AI/AN and non-Hispanic White people. Greater efforts in reducing obesity are needed for AI/AN populations in almost all IHS regions.

### Limitations

Our study has several limitations. First, BRFSS data are self-reported and may be subject to recall and social-desirability bias. Second, although BRFSS oversampled AI/AN people in 11 states in 2017, the overall sample size of AI/AN people remains small, and some of the estimates (especially for heavy drinking) were unstable or unreportable. Third, for current cigarette smoking, BRFSS did not inquire via the core questions about the types of cigarette smoked; in particular, no information was collected on the traditional tobacco products used in Indigenous populations. Fourth, data on physical activity included only leisure-time physical activities and did not consider other forms of physical activity, such as active transportation or jobs with a high demand for energy expenditure. Lastly, although this study demonstrated that significant regional disparities existed in health-related behavioral risk factors and obesity among AI/AN people after considering individual-level sociodemographic characteristics, because of lack of information we were unable to explore the reasons why some health-related risk factors may be more or less severe in one region than in other regions. Future research is warranted to examine the trends of health-related risk factors over time in all IHS regions to help refine public health programs that focus on AI/AN individuals. Multi-level analyses can be conducted to further examine the regional factors including cultural and socioeconomic determinants, as well as previous or ongoing public health intervention programs, that may contribute to regional disparities when information is available.

### Conclusion

This study expands on scientific knowledge exploring racial and ethnic disparities, specifically in AI/AN people in health-related behavioral risk factors and obesity. Our analysis of population-based surveillance data demonstrated that AI/AN people continue to experience a greater burden of obesity and behavioral risk factors, especially current smoking, than non-Hispanic White people at the nationwide level as well as by IHS region. The significant disparities across IHS regions that we found provide a strong evidence base for tribal communities. Our findings support the importance of public health efforts to address and improve these risk factors in AI/AN people through culturally appropriate interventions. As the programs for chronic disease prevention and intervention among AI/AN populations continue, our findings can be applied to future intervention program planning and also serve as references for key measures for program evaluation. Moreover, prevention and intervention programs may be designed within an ecologic perspective that incorporates family relations, neighborhood environment, and culturally appropriate dissemination of information to enhance psychosocial traits (eg, attitudes, knowledge, intentions) toward healthier lifestyles in AI/AN communities.
